# Screening and validation of genome-edited animals

**DOI:** 10.1177/00236772211016922

**Published:** 2021-07-01

**Authors:** Rosie K Bunton-Stasyshyn, Gemma F Codner, Lydia Teboul

**Affiliations:** The Mary Lyon Centre, Medical Research Council Harwell Institute, UK

**Keywords:** GM, Quality assurance / control, Animal model, PCR

## Abstract

The emergence of an array of genome-editing tools in recent years has facilitated the introduction of genetic modifications directly into the embryo, increasing the ease, efficiency and catalogue of alleles accessible to researchers across a range of species. Bypassing the requirement for a selection cassette and resulting in a broad range of outcomes besides the desired allele, genome editing has altered the allele validation process both temporally and technically. Whereas traditional gene targeting relies upon selection and allows allele validation at the embryonic stem cell modification stage, screening for the presence of the intended allele now occurs in the (frequently mosaic) founder animals. Final confirmation of the edited allele can only take place at the subsequent G1 generation and the validation strategy must differentiate the desired allele from a range of unintended outcomes. Here we present some of the challenges posed by gene editing, strategies for validation and considerations for animal colony management.

## Résumé

L'émergence ces dernières années d'une gamme d'outils d'édition du génome a facilité l'introduction de modifications génétiques directement dans l'embryon, augmentant ainsi la facilité, l'efficacité et le catalogue des allèles accessibles aux chercheurs dans une gamme d'espèces. En contournant l'exigence d'une cassette de sélection et en permettant une large gamme de résultats outre l'allèle désiré, l'édition du génome a modifié le processus de validation d'allèle à la fois temporellement et techniquement. Alors que le ciblage traditionnel des gènes repose sur la sélection et permet une validation d'allèle au stade de la modification des cellules souches embryonnaires, le dépistage de la présence de l'allèle prévu se produit maintenant chez les animaux fondateurs (qui sont souvent mosaïques). La confirmation finale de l'allèle modifié ne peut avoir lieu qu'à la génération suivante (G1) et la stratégie de validation doit différencier l'allèle désiré d'une gamme de résultats inattendus. Nous présentons ici quelques-uns des défis posés par l'édition génétique, les stratégies de validation et les considérations pour la gestion des colonies animales.

## Abstract (de)

Die Entwicklung einer Reihe von Tools zur Genom-Editierung hat in den letzten Jahren die Einführung von genetischen Modifikationen direkt in den Embryo begünstigt und die Einfachheit, Effizienz und den Katalog der Allele, die den Forschern über verschiedene Arten zugänglich sind, erhöht. Das Genome Editing umgeht die Notwendigkeit einer Selektionskassette und führt zu einer breiten Palette von Ergebnissen neben dem gewünschten Allel. Dadurch hat sich der Prozess der Allel-Validierung sowohl zeitlich als auch technisch verändert. Während das traditionelle Gen-Targeting auf Selektion beruht und eine Allel-Validierung im Stadium der ES-Zell-Modifikation ermöglicht, erfolgt das Screening auf das Vorhandensein des gewünschten Allels nun in den (häufig mosaischen) Gründertieren. Die endgültige Bestätigung des editierten Allels kann erst in der nachfolgenden G1-Generation erfolgen, und die Validierungsstrategie muss das gewünschte Allel von einer Reihe von unbeabsichtigten Ergebnissen unterscheiden. Hier stellen wir einige der Herausforderungen im Zusammenhang mit Gen-Editierung, Strategien zur Validierung und Überlegungen zum Management von Tierkolonien vor.

## Resumen

La emergencia en los últimos años de una serie de herramientas de modificación del genoma ha facilitado la introducción de modificaciones genéticas directamente en el embrión, aumentando así la facilidad, la eficiencia y las opciones de alelos accesibles para los investigadores en una gran variedad de especies. Al poder saltarnos la necesidad de un grupo de selección cerrado y con la posibilidad de una amplia gama de resultados además del alelo deseado, la modificación del genoma ha alterado el proceso de validación de alelos tanto temporal como técnicamente. Mientras que la selección tradicional de genes depende de la selección y permite la validación de alelos en la fase de modificación de células madre, hacer un cribado para identificar la presencia del alelo deseado ahora ocurre con los animales fundadores (normalmente mosaico). La confirmación final del alelo modificado solo puede hacerse en la siguiente generación G1 y la estrategia de validación debe diferenciar el alelo requerido de una serie de resultados no intencionados. En este estudio presentamos algunos de los retos que supone la edición genética, así como estrategias de validación y consideraciones para la gestión de colonias de animales.

## From traditional gene targeting to genome editing

### Traditional gene targeting in embryonic stem cells

**Figure 1.**
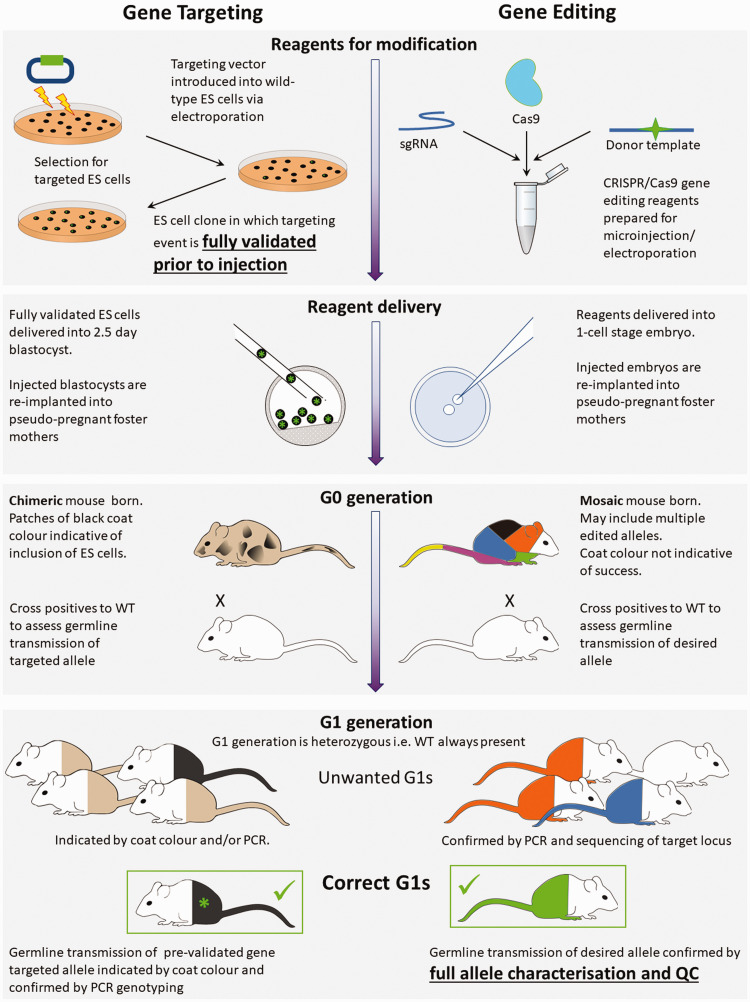
Gene targeting and genome editing processes. Comparison between traditional gene targeting and more recent gene-editing processes from reagents to correct G1 animals. The figure highlights the validation of embryonic stem (ES) cells prior to delivery into the embryo, whereas gene editing technologies rely on validation at the mouse stage. Left: in traditional gene targeting, validation occurs in embryonic stem (ES) cells, prior to their delivery into 2.5-day blastocysts. G0 animals are chimeric, being composed of two pre-defined cell types, those of the host and the validated ES cell. At the G1 generation there are only two possible genotypes to identify. ES cell and host with different coat colours can be used so that coat colour indicates incorporation of the ES cells. At G0 coat colour will be mixed at different ratios depending upon inclusion of ES cells into the embryo. At G1 full coat colour can demonstrate that ES cells have populated the germ-cells of the G0 parent and PCR genotyping can confirm which of the two ES cell derived alleles has been transmitted. Right: in gene editing, genome modification happens *in vivo* after reagents are delivered to the 1-cell stage embryo. G0 mice are mosaic, being composed of cells with multiple different genotypes. Multiple editing events during early embryonic development may produce an assortment of cell-lineages all with differing, and previously undefined, genotypes. At the G1 generation offspring with many different genotypes may be born and it is only at this stage that the desired allele can be definitively identified, and the mutation and background be fully validated. Coat colour cannot be used to indicate success of gene editing as there is no host-donor chimera. PCR, polymerase chain reaction; WT, wild type.

Traditional genome engineering starts with embryonic stem (ES) cells, which are able to populate all cell lineages of the mouse embryo, including the germinal cells.^1^ This method relies upon homologous recombination – the capacity of these cells to recombine exogenous DNA into their own genome in a targeted fashion – without the requirement of additional effectors.^2^ Along with the desired modification, a positive selection cassette is almost always included, to allow for the drug selection of ES cells that have incorporated the targeting vector into their genome. After this initial selection, ES cell clones are screened to identify those in which precise integration of the vector sequence into the target gene has been achieved. The presence of the selection cassette or other novel sequences, such as reporter cassettes, provides unique identifiers to detect clones in which insertion is on target and modification of the target gene has been achieved. Importantly, full molecular characterization of the engineered allele takes place in clonal ES cell populations prior to their microinjection into embryos. Using these techniques, ES cell targeting efficiencies ranging from less than 1% to up to 50% are generally achieved.^3,4^ However, even once the desired allele is generated in ES cell clones, success still depends on the ES cells achieving germline transmission (GLT) (see Figure 1 for a description of a typical gene targeting process).

Successfully modified ES cell clones are injected into host blastocysts and the resultant chimeric G0 offspring are screened for incorporation of clone-derived coat colour/modifications. Positive G0 mice are then mated with wild-type (WT) animals and the resulting G1 progeny are assessed for GLT of the engineered allele; positive animals are then taken forward to establish the new line. Dependency on ES cells meant that genetic engineering was restricted to a small number of mouse strains (mostly 129 and C57BL6/N).

### Genome editing

Genome editing is a new technology based on customizable targeted nucleases, such as zinc finger nucleases,^5^ TALENS^6^ or CRISPR–Cas9.^7^ The use of these molecular tools has revolutionized our ability to modify genomic sequences (as discussed further by Troder and Zevnik^8^).The introduction of specific mutations using this technology still utilizes the innate capacity of the cell to recombine an exogenous DNA molecule (supplied by the researcher) into a homologous region of the genome, but the frequency of recombination has been increased by the introduction of a double-stranded DNA (dsDNA) break (DSB) at the target locus. Efficiency has been increased to such an extent that selectable markers are no longer required, and it is now feasible, indeed commonplace, to carry out gene targeting directly into one- or two-cell embryos instead of in ES cells (see Figure 1). The most recent generation of genome-editing tools (for example, prime-editing and base editing, as discussed by Caso and Davies^9^)^10,11^ only involve single-stranded DNA breaks, but ultimately require similar strategies for genetic validation.

#### Output of editing: mosaic animals

The animals that are born from embryonic editing are very often mosaic: they contain a mixture of cells with different genotypes, probably as a result of nuclease activity and gene editing occurring after the one-cell embryo stage,^12,13^ but also possibly because the DNA break is carried over through cell division.^14^ Genotyping of traditionally derived G0 chimeric mice involved identifying the presence of two predefined genotypes (that of the WT host embryo or that of the fully characterized engineered ES cells). Genotyping of embryonically targeted G0 mosaic animals is often more complicated, as they can often include more than two alleles. In addition, the precise identities of the mutant alleles are not known prior to genotyping and can be represented in unpredictable proportions. Strategies to avoid mosaicism are being developed, but a definitive solution has not yet been identified.^15^ This presents a novel challenge compared to that of screening ES cell colonies and we shall discuss strategies to deal with this.

#### Accessibility and broadening the range of alleles

The ability to create precise edits directly *in vivo* has also released researchers from their previous dependence on robust cultured pluripotent ES cells with retained capacity to reconstitute an embryo. This has opened up the field of genome engineering – once the domain of the mouse – and it is now possible to edit with precision the genomes of a much broader range of laboratory animals, such as *Caenorhabditis elegans*,^16^ squid,^17^ non-human primates^18^ and even humans.^19^

**Figure 2.**
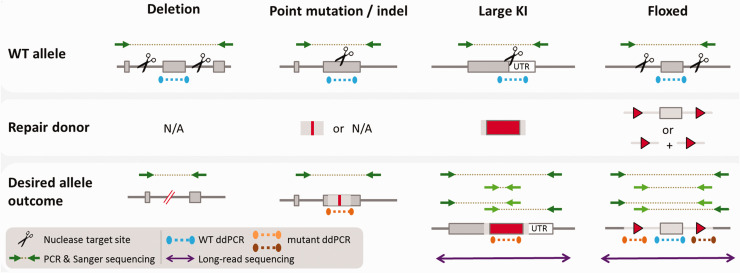
Editing strategies and assays for allele validation. *Deletion:* nucleases target either side of an exon or other region to be deleted. Polymerase chain reaction (PCR) primers flank targeted region and can detect a reduction in amplicon size after deletion. Sequence of the PCR amplicon should be confirmed via Sanger sequencing (or similar). Droplet digital (dd)PCR copy counting of the wild-type (WT) allele (blue assay) in G1s will identify a copy number of one. *Point mutations and indels:* a nuclease is targeted to a single location where the nucleotide change is to be made. PCR primers flank this location. A size shift will not be present in the desired mutant. The nucleotide change must be identified by Sanger sequencing of the amplicon. ddPCR copy counting of the WT allele (blue assay) in G1s will identify a copy number of one. When a repair donor is used to produce a specific mutation, ddPCR copy counting of the mutant sequence (orange) in G1s should give copy number of one. *Large knock-ins:* a nuclease is targeted to a single site for insertion of the knock-in. PCR primers flanking the target location (dark green) can be used to detect a size increase in the presence of an insertion. Primers specific to the repair template (light green) can detect donor insertion. Primer pairs combining one primer binding within the repair donor and another binding outside of the repair donor (light and dark green pairs) can identify on-target donor insertion. Sanger sequencing of these amplicons must be used to confirm identity and may require multiple Sanger reads depending on the insertion size. Long-read sequencing (purple) can identify the entire segment in a single read and confirm whether a fully correct allele is present in the G0 generation. ddPCR copy counting of both the WT allele (blue assay) and the repair donor (orange) in G1s should each give copy number of one. *Floxed:* nucleases target either side of an exon (or other region) where LoxP sites are to be inserted. PCR primers flanking the entire region (dark green) will amplify a larger product, but the ability to discriminate a size shift via standard agarose gel electrophoresis will depend on the relative size of the floxed region. A primer pair specific to the two LoxP insertions (light green) can identify insertion of a single long donor template, or in the case of two short donors, in cis insertion of both donors. Primer pairs combining one primer specific to a LoxP insertion (light green) and another within the flanking target locus (dark green) can identify on target integration. Sanger sequencing of these amplicons must be used to confirm identity and may require multiple Sanger reads depending on the insertion size. Long-read sequencing (purple) can identify the entire segment in a single read and confirm whether a fully correct allele is present in the G0 generation. ddPCR copy counting of the WT allele (blue assay) in G1s will identify a copy number of two. Assays specific to each LoxP insertion (orange and brown) will each give copy number of one. UTR, untranslated region.

Increased efficiency of targeting and the redundancy of selection cassettes has expanded the repertoire of potential genetic changes accessible to genome engineers. It is now possible to engineer categories of changes in a given gene, such as indels,^20^ point mutations^7^ and larger knock-ins (KIs, tags, reporter genes or recombinase coding sequence)^21^ in a completely seamless fashion (Figure 2 and Table 1). The assembly of more complex alleles such as conditional knock-out (KO) or conditionally activatable alleles is also increasingly easier. Indeed, whole regions of chromosomes can be modified to create large-scale deletions, inversions or duplications.^22^ These diverse genetic modifications permit the analysis of gene function or mimic genetic variations that may be causative of disease. Animals with such modifications can be used as pre-clinical models to establish and evaluate therapeutic strategies.

**Table 1.** Description of assays applied at different generational stages when producing gene-edited animals. The expected outcomes at G0 and G1 stages are detailed for correctly edited animals with the assays applied. Refer to Figure 2 for visual representation of the described assays.

Generation	Method	Allele type			
		Deletion	Indel and point mutation	Tag/Large KI	Floxed
G0	**On target PCR and Sanger sequencing** PCR spanning target region. If required, sequence confirmation of allele in both directions.	Reduced amplicon size compared to WT control. Allele sequence to be confirmed by sequencing if specific segment to be excised/deletion too small to be visualized.	Amplicons of equivalent size to WT control. Allele sequence to be confirmed by sequencing.	Larger amplicons compared to WT control. PCR assays can also be designed to: • identify donor insertion (light green primer pair) • Specifically amplify on-target donor insertion (paired light and dark green primers) Allele sequence to be confirmed by sequencing.	Amplicon compared to WT control, depending on relative size of floxed region size difference may or may not be discernible by agarose gel electrophoresis. PCR assays can also be designed to identify: • donor insertion and presence of both LoxP sites in cis (light green primer pair) • on-target donor insertion (paired light and dark green primers) Allele sequence to be confirmed by sequencing.
	**Long read sequencing** Deep interrogation of allele sequence using large number of single reads across large interval. Detection of upstream/downstream events/re-arrangements.^a^	N/A^a^	N/A^a^	Identification of discrete alleles in mosaic animals, including discrimination of features (e.g. LoxP sites) in cis or trans of one another. Confirmation of repetitive regions of allele sequence that cannot be resolved using Sanger sequencing.	Identification of discrete alleles in mosaic animals, including discrimination of features (e.g. LoxP sites) in cis or trans of one another. Confirmation of repetitive regions of allele sequence that cannot be resolved using Sanger sequencing.
G1 (G0 × WT)	**On-target PCR and Sanger sequencing** PCR spanning target region. Sequence confirmation of allele in both directions.	Same PCR assays as for G0. Transmitted allele sequence confirmed by sequencing.	Same PCR assays as for G0. Transmitted allele sequence to be confirmed by sequencing.	Same PCR assays as for G0. Transmitted allele sequence to be confirmed by sequencing.	Same PCR assays as for G0. Transmitted allele sequence to be confirmed by sequencing.
	**Off-target PCR and Sanger sequencing**	PCR spanning off-target site(s). Sequence confirmation of allele in both directions. No evidence of off-target activity detected, i.e. no difference in amplicon size and sequence is WT.			
	**ddPCR copy counting** Copy counting of excised region/insertion sites and HDR donor templates if used to check for random integrations.	Copy counting of the excised region shows copy number of 1.	Copy counting of correct G1 should give copy numbers: Mutant = 1 copy, WT = 1 copy	Copy counting of correct G1 should give copy numbers: Mutant = 1 copy, WT (insertion point) = 1 copy	Copy counting of correct G1 should give copy numbers: 5′ LoxP site = 1, Floxed exon = 2, 3′ LoxP site = 1
	**Long read sequencing** Deep interrogation of allele sequence using large number of single reads across large interval. Detection of upstream/downstream events/re-arrangements.^a^			Confirmation of repetitive regions of allele sequence that cannot be resolved using Sanger sequencing. Entire interval should be covered without error.	Confirmation of repetitive regions of allele sequence that cannot be resolved using Sanger sequencing. Entire interval should be covered without error.

HDR, homology directed repair; PCR, polymerase chain reaction; ddPCR, droplet digital PCR; WT, wild-type;

a Short-read next generation sequencing can be employed.

The repertoire of accessible mutations is ever expanding as methodologies develop and protocols are finessed. Targeted insertions of over 4 kb delivered directly into mouse embryos are becoming increasingly common,^23–25^ with instances of targeted integration of up to 25 kb reported in the literature.^26^ However, some designs remain mostly inaccessible to generation by genome editing directly in embryos. For example, larger or more complex insertions (e.g. KI of *Cre-ERT2*) can be challenging. Equally demanding is the targeting of genes or loci that may be less amenable to editing, possibly due to a lack of nuclease recognition sites, or because the region is physically inaccessible (for example, in a repressed chromatin state) in the early embryo. Those more challenging alleles remain the domain of genetic manipulation in ES cells, sometimes with the assistance of genome-editing nucleases for additional efficiency.

#### Validation comes later in the process: G0 screening and G1 full validation

The advent of genome editing has fundamentally changed the process of validation as new alleles are engineered *in vivo* rather than in cultured cells (Figure 1). With classical homologous recombination, genetic changes can be entirely validated using materials produced *in vitro* and the changes to be assessed are generally clonal in nature and therefore genetically homogenous. Only after full molecular validation are the genetically altered ES cells introduced into embryos to produce animals (Figure 1, left panel).

By contrast, editing directly into embryos naturally precludes the ability to pre-screen before the generation of live animals. Drug-selectable markers are no longer needed in a targeting vector/repair template. This means that genotyping and quality control can no longer rely on these conveniently generic molecular ‘anchors’ but instead must be more tailored and able to detect more subtle mutations. In addition, genome editing directly into early embryos produces mosaic G0 animals containing several alleles of the modified gene (Figure 1, right panel). As a consequence, the validation is a much more complex molecular exercise, which must disentangle the sequences of the different allelic variants that are present in each founder animal. This is made all the more difficult because only small amounts of sample biopsy are available for small laboratory animals, such as a fin biopsy in fish or a small earclip in rodents. Biopsies may not be fully representative of the genetic make-up of the animal overall; they may show both identities and distribution of alleles that are different to those in the germline lineages.^27^ In addition, these analyses may have to be completed quickly if founder animals show welfare issues. For these reasons, G0 screening aims only to identify *potential* founders carrying the desired mutant allele, rather than a full characterization of the edited alleles.

Further screening and full validation can occur at the G1 generation, produced by crossing potential G0 founders to WT mates. At G1, the individual edited alleles present within the G0 founders’ germlines segregate, so that G1 offspring are heterozygous, carrying one WT allele and one allele from the G0 founder. G1 siblings can each inherit a different allele from the G0 parent, so each individual must be screened and fully validated, and can go on to establish a unique mutant line.

### New challenges and new assays

A major challenge in the validation of alleles in edited animals is the characterization of the complex and potentially unexpected outcomes of genome editing, both at the intended target site and potentially at other sites throughout the genome. This is a tall order and requires the application of a combination of molecular assays to differentiate between the expected, and a plethora of unexpected, DNA sequences in order to achieve the full validation of animals. What is more, the initial generation (G0 founder animals) can only be screened for the presence of desired genetic changes in a biopsy taken from them.^13^ Definitive validation of the genetic changes must await the availability of biopsies from the non-mosaic animals at the subsequent generation.

In the following sections we shall describe the methods for generation, screening and validation of engineered alleles produced using CRISPR–Cas9. We shall focus on our process used for production and validation of mutant mice; however, concepts and methods described should be applicable to nuclease-aided genome editing in other model species.

## Genomic validation pipelines for different types of modification

The specific strategy for screening and validation depends on the category of allele engineered, as the initial aim of the exercise is to confirm the presence of the desired allele. The validation process involves screening of G0 animals for the presence of the desired allele, followed by genotyping and definitive validation of the transmitted allele in G1 animals.^13^ Full validation of animals requires more than one assay, in order to detect the desired sequence change and to exclude other changes. Tools used in any standard pipeline include polymerase chain reaction (PCR) with gel electrophoresis, Sanger sequencing of PCR products^7,13,28^ and copy counting assays (for example, droplet digital PCR (ddPCR)^22,28,29^ or quantitative PCR^30^) to quantify the copy number of mutant and WT alleles. For analysis of Sanger sequencing data from G0 animals, online tools (for example in Hsiau et al.^31^) can be very helpful in disentangling peak-on-peak reads and in helping to quantify allele contribution but manual inspection remains desirable for final sign-off on allele validation.

**Figure 3.**
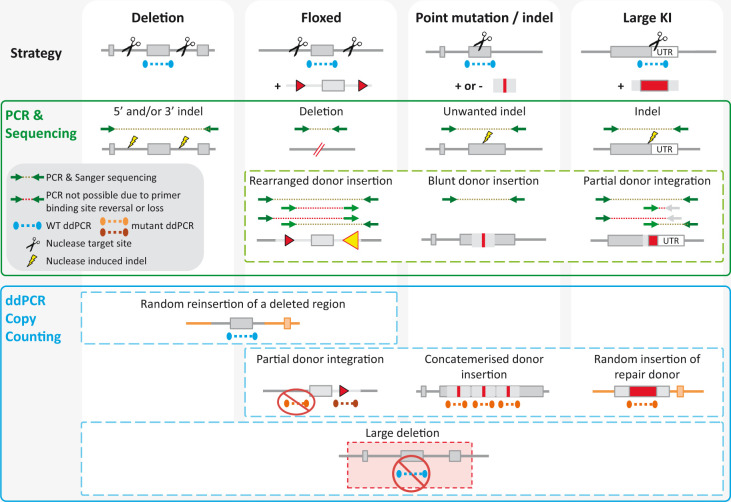
Unintended mutations and methods to detect them. Different types of unintended mutation can occur depending on the editing strategy employed – the number of nuclease target cut sites and whether a repair template is included – while other mutations, such as large deletions, can occur in all cases. Here we present some common examples along with assays which can be used for their detection. Most simply, polymerase chain reaction (PCR) amplification of the targeted locus may identify unwanted insertions or deletion by a shift in band size. Sequencing PCR amplicons can reveal unintended indels and incorrect donor insertions. An inability to amplify an expected product (indicated by a red dotted line) may indicate a rearranged donor insertion (causing incompatible primer orientation) or a partial integration (failure to insert the primer binding region). Copy counting assays, using droplet digital (dd)PCR (or qPCR) are useful for identifying insertion events which are not readily detected by regular PCR. For example, a repair donor or a deleted region can reinsert randomly elsewhere in the genome and ddPCR assays can help to detect this. Concatemerized on target insertion of a donor can be challenging to identify by regular PCR due to amplification bias, while deletions which expand beyond the primers binding sites will be missed entirely. Both are readily detected by ddPCR.

Figure 2 illustrates both the editing strategy for different types of allele and the assays used to detect the desired modified allele. Table 1 summarizes those strategies for detection of deletion, point mutation, tag/cassette KI and floxed alleles and the expected results of applying these assays in correctly edited animals at the G0 and G1 generation. Figure 3 shows some of the non-conforming alleles that can occur in these projects and illustrates how different assays are needed to detect various unwanted outcomes.

### Deletions

The generation of deletions typically employs one or two nuclease target sites on either side of the segment to be deleted. A single-stranded oligonucleotide (ssODN) template DNA is included for the deletion of a defined fragment.

G0 identification of potential founders is generally performed initially by PCR to detect a shift in band size. Deletion boundaries are subsequently confirmed by Sanger sequencing.

G1 validation involves the same strategy. PCR and Sanger sequencing are used to check potential off-target sites near the target locus. Reintegration of the deleted segment should also be excluded; for example, by employing a quantitative PCR method, such as ddPCR or qPCR. For the cases in which a template donor is used, the number of integrated copies is also evaluated by a quantitative PCR method. It is important to note that genome editing can result in the deletion of large intervals, which may result in the loss of the sequences that are recognized by the genotyping primers. In these instances, standard PCR will not result in any band amplification and the deletion will not be revealed by the assay.

### Indels and point mutations

The generation of such alleles typically requires the use of a nuclease for one site, and a repair template (ssODN) for the cases in which a specific indel or a point mutation is required.^27^

G0 identification of potential founders is generally performed by PCR and Sanger sequencing, but it also can be extended to a large-scale process with next generation sequencing.^32^ The validation of the new allele takes place at the subsequent generation and again generally relies on PCR and Sanger sequencing of both the targeted locus and chromosomally linked potential off-target sites. For the cases in which a template donor is employed, the number of integrated template copies is evaluated by a quantitative PCR method, such as ddPCR or qPCR.

### Larger KIs (tags, reporters or Cre coding sequence) and floxed alleles

The insertion of a DNA cassette typically employs one or two nuclease target sites and template DNA, which can be long single-stranded DNA or a double-stranded linear or circular DNA template for larger cargos. In the case of floxed alleles, the DNA template can also be two ssODN templates that correspond to the loxP insertions.

The identification of potential founders generally begins with several PCR assays. One assay specifically amplifies the mutant DNA template, a second assay employs primers anchored on either side of the region to be modified, outside of the donor template. A further two assays are each anchored at a sequence that is specific to the donor and a sequence outside of the donor (see Figure 2). If these assays show the correct profile, the allele quality is subsequently confirmed by Sanger sequencing. It is now possible to validate the longer segments of DNA necessary for larger KIs directly in mosaic animals with new types of molecular assays (see section ‘Other techniques, old and new, to validate the identity of larger genetic segments’, below).

The definitive validation of the new allele, which takes place at the subsequent generation, involves the same strategy. PCR and Sanger sequencing are used to check chromosomally linked potential off-target sites. The number of integrated copies of the template donor(s) is also evaluated by a quantitative PCR method.

### Screening for off-target activity – all allele types

For many animals that are modified by genome editing, it is only essential to check potential off-target sites that are chromosomally linked to the target locus, as other unwanted modifications can be bred out by breeding to WT stock. The potential off-target sites can be identified using many online search engines (for example, Concordet and Haeussler^33^ and Hodgkins et al.^34^). We typically find and check those with two or fewer mismatches with the target sequence, focusing in particular on mismatches outside of the seed sequence (that is, the most important part of a sgRNA to direct its sequence specificity) when using CRISPR–Cas9. These potential off-target sites are screened by PCR amplification and Sanger sequencing and, if resources are available, by ddPCR to identify potential larger deletions.

These assays are carried out at the G1 generation; however, for the cases in which backcrossing to WT animals to produce a G1 generation is impractical or too costly (for example, in the case of large animals), it may be preferable to widen off-target checks to all those that can be identified or captured in the genome, by using a next generation sequencing-based method.

## Other techniques, old and new, to validate the identity of larger genetic segments

Alleles with a larger modified DNA interval or a more complex structure require assays that interrogate longer segments and/or a broader combination of assays. Some of these include classical techniques such as Southern blotting,^35^ whereas other are much more recent, such as long-range sequencing, examples of which include Nanopore^36^ and PacBio.^37^ The latter are particularly useful in checking the entirety of new alleles encompassed in a single read, thus identifying whether discrete sequence changes are clustered in *cis* on the same allele or sit in *trans* of one another. Other methods that rely on *in-situ* hybridization analyse large-scale chromosomal changes instead of interrogating the sequence of a given DNA segment (for example, fiber-fluorescence *in-situ* hybridization^38^). Sequencing of the whole genome seems to be the ultimate solution to complete validation of genome-editing outcome and has been used for the analysis of *in-vivo* CRISPR–Cas9 activity, but the data are particularly difficult to understand in mosaic animals.^39^ Whichever assays are employed, the analysis of these very large datasets remains a complex exercise that requires awareness of the variety of events that may arise from nuclease activity.^40^ All of these methods are labour intensive and expensive. They also require specialist molecular biology and/or bioinformatic skills and are less scalable to the analysis of many animals.

## You don’t always get what you want: failure and complexity of outcome

Current genome-editing tools target genetic changes to a given locus through generation of a DSB or modification of bases but do not guarantee that the intended sequence is the outcome of editing. Instead, genome editing relies on one of many endogenous pathways of DNA repair. As a consequence, many unwanted allelic variants are also generated in the process of genome editing (see examples in Figure 3).

### On-target cutting can result in failure to generate a desired allele

In parallel with the desired genetic modifications, other outcomes can occur in other cells of the same founder animal. For example, in-frame indels may occur when a frame shift is required.^7^ Silent mutations^27^ and even gene conversion^41^ (a repair event in which the other endogenous allele acts as a repair template) can be examples of other unintended outcomes. In addition, DNA segments excised by the nucleases (or other sequences) can insert into the genome in an uncontrolled manner, or undesired insertions can occur at the nuclease target site during the repair event. By contrast, fragments much larger than the segment flanked by the nuclease recognition sites can be deleted (even from a single cut site).^42–44^

### Imperfect or additional insertions

A donor molecule can be integrated in a partial or rearranged fashion, or even as a concatemer of several copies, to generate an unwanted new allele at the target site^13,28,45^ and/or at other genomic locations (see below; off-target sites). Donor integration can be a result of blunt insertion rather than the product of seamless homologous recombination. Excised regions (or part of these) can be re-inserted, sometimes in combination with other, unrelated, DNA sequences. The integrated segments can be as large as several tens of kilobases.^22,38^

### Off-target sequence changes

It was demonstrated early in their utilization that genome-editing nucleases can also generate cuts in DNA sequences other than the intended target. These sequences can differ from those of the intended targets by several bases. The outcome of this off-target activity can be any of the whole range of sequence variations that are described for on-target changes; for example, indels and large deletions or insertions of endogenous or donor sequences.^13,28,44^ Thankfully, with appropriate experimental design, such events are significantly less common than on-target sequence changes, but their occurrence still must be investigated when full validation of genome-edited animals is carried out.^39^ Importantly, any DNA sequences introduced in the zygote, including plasmid sequences for the expression of nucleases or carrying donor templates, can be unintentionally integrated in a random fashion,^40^ probably by the mechanism exploited in classical additive transgenesis.

### Alleles detected in G0s can be different to those validated in G1 animals

As these animals are mosaic, alleles identified in G0 founder animals may not transmit to the G1 progeny. Conversely, some alleles can be found in G1 animals that were not detected during analysis of their G0 parents, either because they were less represented in the founder biopsy,^27^ or because they were masked by other alleles that were preferentially detected by the molecular assays used (for example, because they generate different sized PCR fragments). Pre-implantation genetic screening that samples a small number of cells may not yield an exhaustive survey of the genetic make-up of embryos.^19^

## Transcriptional and post-transcriptional validation

Even when a thorough genomic validation has identified an animal with the precise desired mutation, this is no guarantee that the edited gene will behave as predicted based upon genome annotations. Alternative splicing and use of alternative open reading frames can result in ‘KO escape’ or other unexpected transcriptional and translational consequences in KO and KI alleles.^46,47^ Many techniques can be used for post-genomic validation of the model; these can be broadly split into either transcriptional or translational analyses.

The simplest method of transcriptional analysis is reverse transcriptase PCR (RT-PCR), often followed by sequencing. These techniques can be used, in combination with quantitative PCR methods, to confirm the presence and level of expression of the intended mutant transcript and to identify unintended transcripts that might use novel splice sites, or that might exclude or include entire exons.^29^^.^^48^ For detection of novel transcriptional start or termination sites, 5′ and 3′ rapid amplification of cDNA ends (RACE) can also be combined with sequencing.^47^ Quantitative RT-PCR techniques such as qPCR and ddPCR can also be useful to assess the loss of transcripts in KO models or to check that transcript abundance is not adversely affected, especially when allele designs include changes to regulatory sequences.^46^^.^^48^ These techniques rely on some prior knowledge of the transcripts to be detected. In cases where an unbiased analysis is required, whole transcriptome RNAseq can be employed.^47,49^

The classical method to identify translational consequences of a mutant allele is the western blot, which uses an antibody to measure protein levels. Antibody specificity can vary widely and so care is required when selecting and testing these reagents. In some cases antibodies may be available that can detect both the WT and mutant or engineered proteins; others will be specific to the WT and it may even be possible to raise an antibody that specifically recognizes only the engineered mutant.^50^ As with PCR-based methods of transcriptional analysis, antibody-based translational analysis relies on prior hypotheses of the translational outcomes of the designed allele and is further limited by the availability of appropriate antibodies. For unbiased analysis, mass spectrometry has been used in high-throughput cellular assays to identify residual protein expression and proteins produced from unexpected splice forms; however, this methodology is not commonly employed in mouse mutant validation.^49^

Also of note is that different KO strategies can result in different phenotypes, even when all cases result in a loss of protein product. This can be due to genetic compensation and adaptive transcription of related genes, which counteract the effect of a gene KO. Consideration of the best design to address the experimental question and quantitative RT-PCR assessment of related genes may be warranted.^51–53^

## Day to day considerations for animal management

### Official nomenclature and in-house nomenclature

Use of official nomenclature of edited alleles^54^ is essential for accurate publication of results and dissemination of reagents, but it is not practical to reflect and track the variability of alleles during the genome-editing process. When generating and breeding genome-edited animals, it is essential to employ a stock nomenclature that can keep up with the evolving genetic make-up of each generation. Mosaic founders give rise to a subsequent generation in which siblings within a litter can carry different alleles; each of these G1 mice can potentially found a different mouse line with a particular variant in the subsequent generation. In-house nomenclature must differentiate the founder generation (generally mosaic), the G1 generation obtained by mating a founder to WT animals (comprising siblings that can each carry a different version of the altered allele), and the subsequent generations in which mutations follow standard Mendelian patterns of transmission.^13^ Tracking of the generation number as well as the lineage is therefore essential to keep track of the genetic make-up, because of the complex genetic constitution of the first two generations. This is fundamentally different to the genetic make-up observed in the traditional ES cell method, in which the founder animals are chimeras that contain a mixture of WT cells and mutated cells, all of which bear the same genetic alteration that was validated *in vitro* prior to the injection of ES cells.

### New kind of welfare continuous assessment

**Figure 4.**
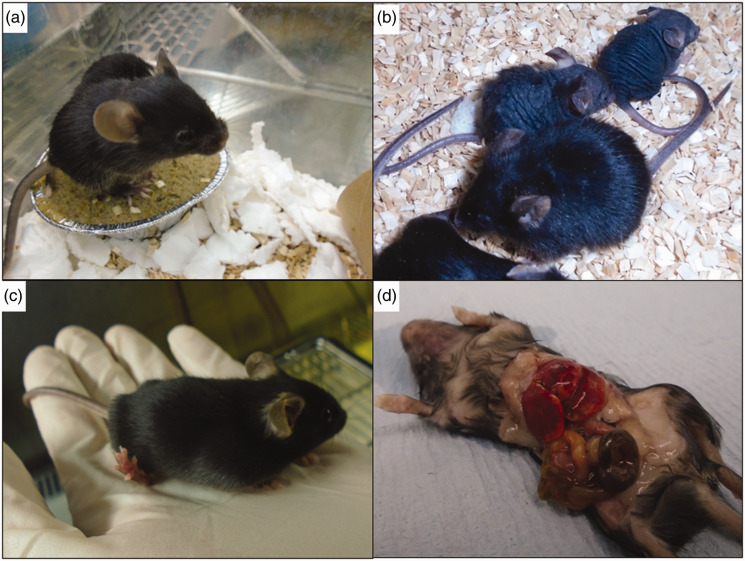
Animals with visual phenotypes. A variety of phenotypes were observed in CRISPR G0 mice from four different projects. (a) Short faces, domed head and missing teeth were observed in 11 out of 24 animals born from a cytoplasmic CRISPR–Cas9 injection to introduce a point mutation into *Csf1r*. (b) Pups with hair loss and/or tufty hair across the whole body were seen in three out of 15 animals derived from a CRISPR–Cas9 pronuclear injection to introduce a point mutation in *Foxn1*. (c) Pup with abnormal hind legs and gait derived from CRISPR–Cas9 electroporation to introduce a point mutation into *Itpr1*. Hind feet point upwards and pup weight bears on hind heels, displaying abnormal movements and hopping. (d) Oedema and odd body shape were observed in all pups born from a cytoplasmic CRISPR–Cas9 injection to introduce a point mutation into *Lemd2*. Upon dissection, the liver was found to be enlarged.

Classical transgenesis by homologous recombination generally involved the injection of ES cells that carried a heterozygous genetic modification, many of which were conditional to cre deletion; this limited the risk of welfare implications in founder animals to rare mutations with dominant effect. Direct genome editing of early embryos represents a different burden of mutagenesis in founder animals, as it is not uncommon that both alleles are modified in the process. Furthermore, these animals often carry several variants of the targeted allele, which populate different cell lineages in an unpredictable fashion. Consequently, phenotypes associated with the function of the gene of interest (and possibly with off-target loci) commonly arise at the founder generation in the process of editing animals (see examples in Figure 4). These founders require particularly intensive welfare observation, for both the phenotypes expected in relation to the project and unexpected phenotypes. Unusually, the subsequent generation may represent less of a challenge in terms of welfare, as the animals return to heterozygosity and, therefore, will only show the effect of dominant alleles.

## Other 3Rs considerations for genome edited animals

Liberation of genome modification from the confines of the mouse house will inevitably increase the range of species used in biomedical research. This has important ethical implications, and careful consideration of the guiding principles of the 3Rs is essential when planning these experiments to evaluate whether the potential benefits of your chosen species outweigh the potential harms (see Davies^55^ for recent review of harm-benefit analysis).

While nuclease mediated gene editing makes genome modification faster and easier, a larger proportion of the work is carried out *in vivo* and the delay of full validation until the G1 generation means that more animals are bred as compared to traditional gene targeting. Since their inception, CRISPR gene editing methodologies have been repeatedly refined, reducing their *in-vivo* burden, for example by aiming to reduce the level of mosaicism or to increase the likelihood of achieving the desired mutation over unintended outcomes.^56,57^ Planning and applying robust and efficient validation protocols, such as those described here, is an important way to minimize unnecessary breeding of research animals. Technological and methodological developments in screening and validation strategies are also moving us closer to being able to validate modified alleles at G0: long-read sequencing, for example, facilitates quantitative assessment of the allelic composition of mosaic animals, even in the case of larger or more complicated modifications making it possible to identify animals harbouring the desired mutation at the G0 generation, reducing the number of founder animals taken forward to breed to G1.^36^

## Conclusion: choose your weapons wisely

In summary, genome editing is a simple concept that has greatly enhanced our ability to manipulate the genetic make-up of animals. However, its implementation remains a challenge, as the technology creates both genetically complex animals and unpredictable genetic changes. It is impossible to fully understand with simple molecular assays the genetic make-up of the new animals that are produced. Furthermore, the assays that are used to identify the presence of the desired outcome must be tailored to the type of intended allele, as again, no single simple assay will address all possible allele types. Only subsequent, in-depth, molecular characterization that is only practical in a small number of animals will yield a full picture of elicited genetic changes. The art of genotyping genetically edited animals is to begin with the molecular assays that are easiest and cheapest to implement, but also to understand their limitations.
